# Prehabilitation in head and neck cancer patients: a literature review

**DOI:** 10.1186/s40463-020-00486-7

**Published:** 2021-01-06

**Authors:** Irene Loewen, Caroline C. Jeffery, Jana Rieger, Gabriela Constantinescu

**Affiliations:** 1 Department of Communication Sciences and Disorders, Faculty of Rehabilitation Medicine, University of Alberta, 8205 114St 2-70 Corbett Hall, Edmonton, Alberta T6R 3T5 Canada; 2 Faculty of Medicine and Dentistry, Division of Otolaryngology – Head and Neck Surgery, University of Alberta, Edmonton, AB Canada; 3 Institute for Reconstructive Sciences in Medicine (iRSM), 1W-02, 16940-87 Avenue Misericordia Community Hospital, Edmonton, Alberta Canada

**Keywords:** Review, Dysphagia, Deglutition, Head and neck cancer, Prehabilitation, Exercise, Preventative, Prophylactic

## Abstract

**Background:**

Dysphagia is one consequence of head and neck cancer that has a significant impact on quality of life for head and neck cancer survivors. While survival rates continue to improve, focus has shifted to maximizing long-term function, with prevention or prehabilitation programs becoming more common. Prehabilitation programs typically include an exercise regime that specifies the exercise type, the number of repetitions to complete per set, the number of sets of each exercise to complete per day, as well as the length of the treatment block. Ideally, exercise programs are designed with principles of neuromuscular plasticity in mind.

**Methods:**

Twenty-nine original research articles published between 2006 and 2020 were included in this state-of-the-art review and examined for program timing and details.

**Results:**

Two definitions for prehabilitation were noted: one third of the studies defined prehabilitation as preventative exercises prior to the start of acute cancer treatment; the remaining two thirds defined prehabilitation as treatment concurrent prehabilitation. Exercises prescribed ranged from general stretching and range of motion exercises, to trismus and swallowing specific exercises. The most common swallowing specific exercise was the Mendelsohn’s maneuver, followed by the effortful swallow, Shaker, and Masako maneuver. The most common dose was 10 repetitions of an exercise, three times per day for the duration of radiation therapy. The most common measures were questionnaires, followed by g-tube dependence, mouth opening, and MBS reports.

**Conclusion:**

This review of the literature has shed light on the variability of prehabilitation timing, exercise type, dose, duration of treatment, and outcomes associated with prehabilitation, making the selection of an optimal prehabilitation program difficult at this time.

## Introduction

Dysphagia or swallowing dysfunction is one of the negative consequences of head and neck cancer (HNC) due to cancer-related destruction of vital structures, treatment side effects, and long-term sequelae or toxicity from systemic therapies such as chemotherapy and radiation [1]. Up to 80% of patients treated for oropharyngeal cancer will have swallowing dysfunction in daily life [1]. As this subtype of HNC increases in incidence, [2, 3] the number of patients needing dysphagia management is also on the rise. In addition, with improved survival rates, there is an increased focus on maximizing long-term function, with prevention being a key approach. An example of this preventative approach is prehabilitation.

Prehabilitation was defined by Silver and Baima [4] as the process in the continuum of care that occurs between the time of cancer diagnosis and the beginning of acute treatment in which targeted interventions are aimed to reduce the incidence and severity of current and future impairments. According to Silver and Baima 2013, [4] prehabilitation is typically used in anticipation of surgery and serves multiple goals, including obtaining a baseline of function, identifying any pre-treatment impairments, addressing overall physical and emotional well-being, and reducing impairments that are expected as a result of treatment. Historically, prehabilitation was first introduced to improve the nutrition and physical ability of military recruits, but healthcare quickly adopted the practice as an opportunity to reduce hospital stays and healthcare costs. Although most prehabilitation studies come from preparing patients for surgery (e.g., orthopedic literature), cancer prehabilitation is emerging as an opportunity to address impairment before it begins. For a more in-depth summary of prehabilitation in cancer patients, and recommendations, the reader is directed to Silver and Baima 2013, [4] Silver et al. 2013 [5] and Silver et al. 2015 [6].

Randomized control trials in cancer populations other than HNC, [7, 8] such as breast cancer and lung cancer, have found significant improvements in post-surgical outcomes when exercises were completed prior to surgery. This principle of prehabilitation is beginning to be applied in dysphagia rehabilitation for HNC patients [4, 6, 9–12]. Prehabilitation can provide HNC patients and their treating clinicians with baseline measures of pre-treatment function and data that may assist with cancer treatment planning. In addition, engaging in prehabilitation before surgery and radiation therapy may provide the opportunity for habit formation, may improve patient knowledge and buy-in of the importance of swallowing exercise, and may improve health and function above baseline status. However, Silver et al. suggest that cancer prehabilitation should be multi-modal in nature and linked to physical exercise, nutritional and psychosocial well-being, and smoking cessation programs [6].

There is variable use of the term *prehabilitation* in the field of dysphagia. While some authors refer to prehabilitation as therapy provided before the start of acute cancer treatment, others use the term to reference therapy provided before the onset of dysfunction. In this review, *pretreatment prehabilitation* will be used to describe preventative exercise programs prior to the start of acute cancer treatment; *treatment concurrent prehabilitation* will be used to describe preventative exercise programs prior to impairment, but beginning at the onset of cancer treatment.

Prehabilitation programs typically include an exercise regimen that specifies the exercise type, the number of repetitions to complete per set, the number of sets of each exercise to complete per day, as well as the length of the treatment block. Ideally, exercise programs are designed with principles of neuromuscular plasticity in mind. Neural plasticity is the brain’s ability to change in response to variations in input from the sensorimotor system. Robbins et al. [12] reviewed ten principles of neural plasticity and integrated these with dysphagia literature for translation into treatment paradigms. In an effort to further support dysphagia rehabilitation, Burkhead et al. [9] summarized principles of neuromuscular plasticity from fields of physical rehabilitation, exercise science, and sports training and related the research in these fields to muscles of mastication and swallowing. Both articles described the importance of the details of an exercise regimen, as these seemingly small features will have differing results to the neuromuscular system. For example, the number of repetitions of an exercise included in a set may differ depending on the goals of the exercise program. A program designed to improve strength and endurance of a muscle requires more repetitions per set (e.g., 8 to 12), whereas a program designed to improve strength and power of a muscle requires fewer repetitions per set (e.g., 6 to 8) [9]. The contributions from these two teams provide essential information to any group looking to implement and justify an exercise program related to dysphagia rehabilitation.

Several studies have described dysphagia prehabilitation in HNC patients, most showing promising results. However, optimal design of a pretreatment exercise regimen is yet to be determined. A literature review of original research articles related to prehabilitation in dysphagia in patients with HNC was carried out to understand the current state of knowledge and identify priorities for future investigation and research [13]. The review was motivated by a need to understand the design of a prehabilitation program, such as exercise types, dose, and treatment block length typically prescribed in dysphagia prehabilitation programs.

## Methods

A state-of-the-art review [13] of the literature was conducted to understand the existing knowledge of prehabilitation in dysphagia. A comprehensive search was conducted in Ovid MEDLINE(R), including Epub Ahead of Print, In-Process & Other Non-Indexed Citations and Daily and Ovid MEDLINE(R) Daily, with publication dates limited to 1990 to 2020 English articles. Search terms included: (prehabilitation OR prophylactic OR preventative OR pretreatment OR prehab OR perioperative) AND (therapy OR rehabilitation) AND (head and neck cancer OR head and neck cancer neoplasm) AND (dysphagia OR deglutition disorders) as free text searches. Only original articles that focused on adult patients were included. Where more than one article was published on the same study, only the article that reported on outcomes of the program was included. Though prehabilitation has differing definitions, both prior to impairment and prior to treatment were included. No exclusions were made based on head and neck subsite or oncologic treatment (single versus multi-modal). Articles related to prophylactic feeding tubes were excluded. Case reports and review articles were excluded, although the reference lists were examined to detect articles that were potentially eligible for inclusion. Reference lists of included articles were also examined to detect articles that were potentially eligible for inclusion.

Once all of the included articles were obtained in full text, they were reviewed to extract the following details: study type (prospective versus retrospective), sample size, point in the treatment continuum when prehabilitation was initiated, type of exercises prescribed, recommended exercise dose, duration of treatment block, justification for exercises and dose, outcome measures, study results, participants, and treatment modality. Data extraction was performed by a single author (IL) who is a speech-language pathologist (SLP) with extensive experience in HNC. Table 1 summarizes the articles included in this review.

**Table 1:** Summary of included articles

First Author	Year	Study Type	Comparator Group	Sample Size by Allocation Group	Baseline Measures of Functional Outcomes	Overall Functional Outcomes
Ahlberg	2011 [14]	Nonselective, longitudinal, prospective cohort study	Alternate RT center	Experimental *n*=190 Control *n*=184	Yes	Negative Equivalent
Ajmani	2018 [38]	Retrospective cohort study	Patients seen prior to initiation of swallowing rehabilitation program	Experimental *n*=119 Control *n*=135	Yes	Positive
Baudelet	2020 [32]	Study protocol for a randomized controlled trial	Service delivery method	Proposed 1:1:1 recruitment App supported *n*=50 Therapist supported *n*=50 Patient directed *n*=50	Yes	N/A
Carmignani	2018 [30]	Part 1: retrospective Part 2: prospective two-arm case–control analysis	Usual care	Experimental *n*=6 Control *n*=6	Yes	Positive
Carnaby-Mann	2012 [15]	Randomized Control Trial	Sham exercises Usual care	Experimental *n*=20 Sham *n*=18 Usual Care *n*=20	Yes	Positive Equivalent
Carroll	2008 [35]	Retrospective case control	Post cancer treatment exercise only	Experimental *n*=9 Control *n*=9	No	Positive Equivalent
Cnossen	2014 [22]	Feasibility study	Mode of program delivery	Leaflet *n*=11 Booklet + DVD *n*=11 Online *n*=11	No	N/A
Cnossen	2017 [24]	Prospective clinical cohort study	None	*n*=50	Yes	N/A
Duarte	2013 [36]	Case series with chart review	Noncompliant group	Experimental *n*=57 Control *n*=28	Yes	Positive Equivalent
Govender	2020 [33]	Two-arm parallel group non-blinded randomized feasibility trial	Usual care	Experimental *n*=16 Control *n*=16	Yes	N/A
Guillen-Sola	2019 [26]	Protocol for a single-blind randomized clinical trial	Late intervention (immediately after CRT completion)	Proposed 1:1 recruitment	Yes	N/A
Hajdu	2019 [27]	Randomized Control Trial	None (reports on adherence within the experimental group of a randomized control trial)	*n*=45	N/A	N/A
Hutcheson	2013 [42]	Retrospective observational study	Non-adherent group	Experimental *n*=286 Control *n*=211	No	Positive
Kotz	2012 [16]	Randomized controlled trial	Usual care	Experimental *n*=13 Control *n*=13	Yes	Positive
Kraaijenga	2015 [23]	Randomized controlled trial	Repeated Measures	Baseline *n*=55 2 year *n*=29 6 year *n*=22	Yes	Positive
Kulbersh	2006 [17]	Prospective cohort study and cross-sectional QOL analysis	Post treatment exercise (first visit after the initiation of their treatment).	Experimental *n*=25 Control *n*=12	No	Positive Equivalent
Mashhour	2018 [28]	Comparative study	Not defined	Experimental *n*=30 Control *n*=30	Yes	Positive Equivalent
Messing	2017 [25]	Randomized controlled trial	Usual care	Experimental *n*=30 Control *n*=30	Yes	Positive Equivalent
Mortensen	2015 [18]	Randomized controlled trial	Usual care	Experimental *n*=22 Control *n*=22	Yes	Equivalent with positive trends
Ohba	2016 [37]	Retrospective case-control design	Exercise provided when dysphagia developed	Experimental *n*=21 Control *n*=30	Yes	Positive
Peng	2015 [40]	Retrospective case series	Prior to initiation of Swallowing Preservation Protocol (SPP) and those not enrolled once SPP was initiated	Experimental *n*=41 Control *n*=66	Yes	Positive
Retel	2011 [41]	Retrospective cost-effectiveness analysis	Usual Care	Experimental *n*=37 Control *n*=53	No	Positive
Shinn	2013 [39]	Retrospective	None	*n*=109	No	N/A
Starmer	2018 [34]	Feasibility trial	None	*n*=36	No	N/A
Tang	2011 [19]	Randomized controlled trial	Usual Care	Experimental *n*=22 Control *n*=21	Yes	Positive
van der Molen	2011 [20]	Randomized controlled trial	Usual Care	Experimental *n*=27 Control *n*=22	Yes	Positive
Virani	2015 [21]	Prospective alternate assignment and characteristic matching	Repetitive swallows	Experimental *n*=26 Control *n*=24	Yes	Positive Equivalent
Wall	2020 [31]	Three-arm prospective stratified parallel group study with 1:1:1 random allocation.	Repeated Measures Service delivery mode	Technology *n*=25 Clinician directed *n*=25 Patient directed *n*=27	Yes	N/A
Wells	2016 [29]	Feasibility study using quantitative and qualitative research methods within a quasi-experimental design	Usual Care	Proposed: Experimental *n*=40 Control *n*=30	Yes	N/A

## Results

Twenty-nine original research articles were included in this review. These articles were published between 2006 and 2020 with an increase in the number of publications on this topic over time, particularly over the last 8 years. Twenty-one of these studies were prospective studies [14–34] and eight were retrospective [35–42]. The sample sizes ranged from twelve [30] to 497 participants [42]. The majority of the sample sizes were small, with 18 studies including 60 or fewer participants [15–25, 27, 28, 30, 33–35, 37].

### Participants and Cancer treatments

Studies included patients with cancer of the oral cavity, oropharynx, hypopharynx, larynx and unknown primary. Approximately half of the articles (*n* =15) made no mention of the HNC etiology; thirteen articles mentioned participants had squamous cell carcinoma; three articles referenced the human papillomavirus (HPV) subset. All studies were based on patients undergoing radiation therapy (RT) or concurrent chemoradiation (CRT) therapy. Six studies [22, 27, 29, 30, 33, 34] included surgical patients; however, the prehabilitation program appeared to be implemented preRT/CRT, not pre-surgery. Three studies [28, 37, 39] did not specify if surgical patients were included.

### Prehabilitation phase and terminology

**Fig. 1:**
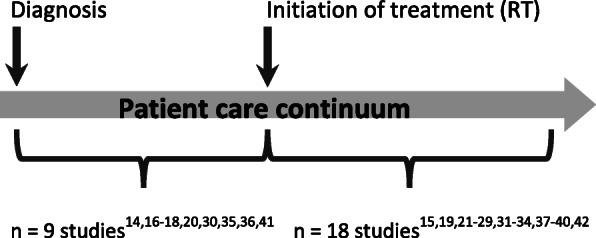
Initiation of prehabilitation programs across the patient care continuum

As the studies were reviewed, it was clear that the term *prehabilitation* was used to refer to different types of programs in the literature. As previously stated, *prehabilitation* was defined by Silver and Baima [4] as the process in the continuum of care that occurs between the time of cancer diagnosis and the beginning of acute treatment. Prehabilitation often involves prescribing prophylactic exercises during the pre-treatment phase of cancer care with the intention to prevent or reduce the severity of anticipated post-treatment dysphagia [4–6]. However, the definition of prehabilitation in the current literature herein varied, with most studies having defined *prehabilitation* as prophylactic exercises completed prior to *impairment* [15, 19, 21–29, 31–34, 37–40, 42] rather than prior to *primary cancer treatment* [14, 16–18, 20, 30, 35, 36, 41]. All studies used the term to imply prevention of impairment as a result of HNC treatments, but some programs began their prehabilitation program prior to the start of cancer treatment, while the majority began theirs at initiation of radiation therapy. Figure 1 shows where in the cancer treatment timeline prehabilitation occurred in the studies included in this review.

### Exercises

**Fig. 2:**
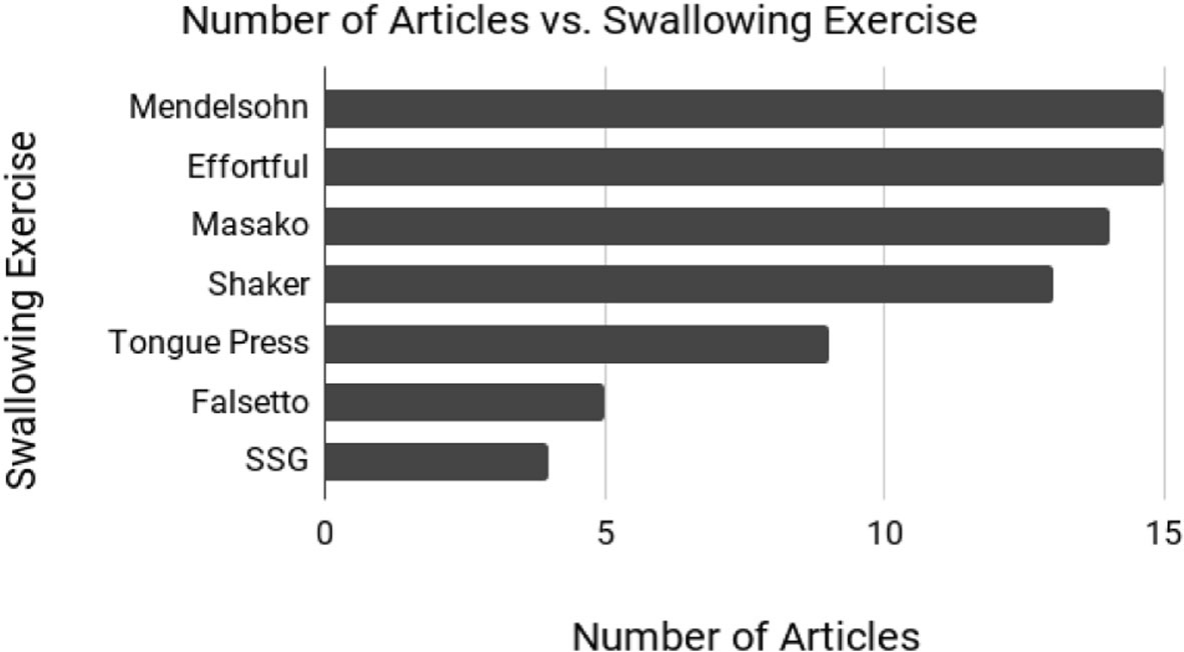
Type of swallowing exercises by the number of articles that included the exercise in their prehabilitation program. *Abbreviations:* SSG = Super Supraglottic Swallow

The types of exercises recommended to patients varied between articles. These included general stretching and range of motion exercises (9 studies), trismus (15 articles), and swallowing specific exercises (22 articles). With respect to the swallowing rehabilitation exercises, Fig. 2 illustrates the distribution of the specific exercises reported in the 22 articles. Seven articles did not define the prescribed exercise regimen [22–24, 26, 28, 33, 41]; however, six of these articles referenced previously developed programs [22–24, 33, 41]. The articles also were reviewed for justification for the exercises assigned in the prehabilitation program. Twenty-one articles provided justification for the exercises assigned in their prehabilitation programs, and of those, ten articles [22, 23, 27, 28, 30, 31, 33, 36, 40, 41] referenced programs previously designed as justification.

### Dose and treatment block

As one of the goals of this review was to understand an optimal design of preoperative exercise interventions, the recommended dose used by each article was examined. In order to note similarities and differences between studies, the following formula was used:

(# of recommended exercises a day) x (# of recommended repetitions a day) x (recommended # of times a day) = number of repetitions, or Dose.

**Fig. 3:**
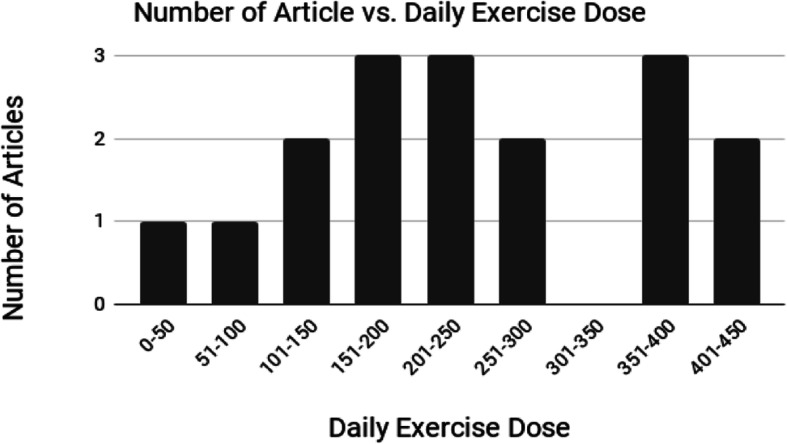
Prescribed daily exercise dose by number of articles that recommended that dose

For example, if a program recommended five exercise types, at ten repetitions each, three times in a day, the calculated dose was 150 (5 × 10 × 3 = 150). Using this calculation when looking at what clinicians and researchers prescribed as part of these prehabilitation programs, the daily dose of exercises recommended ranged from 30 to 440, with the most commonly prescribed dose being 160 repetitions a day. However, one article reported on the number of trials completed by participants, rather than that recommended [22] and one article customized the prehabilitation program to each individual client [27]. If including these articles that have a range due to reporting the number of trials completed by participants or variability in recommended dose based on individual participants, the range of exercises becomes 4–504. Most studies did recommend that the total dose be spread throughout the day. Only three of the studies [20, 21, 37] provided justification for the dose chosen in their prehabilitation program. Figure 3 shows the number of studies recommending a daily dose of exercises.

Duration of the treatment block was defined in 15 studies [14–16, 21–27, 30–32, 36, 37]. The most common recommendation was the duration of radiotherapy [15, 16, 21, 22, 27, 30, 31, 37].

### Outcome measures and treatment results

**Fig. 4:**
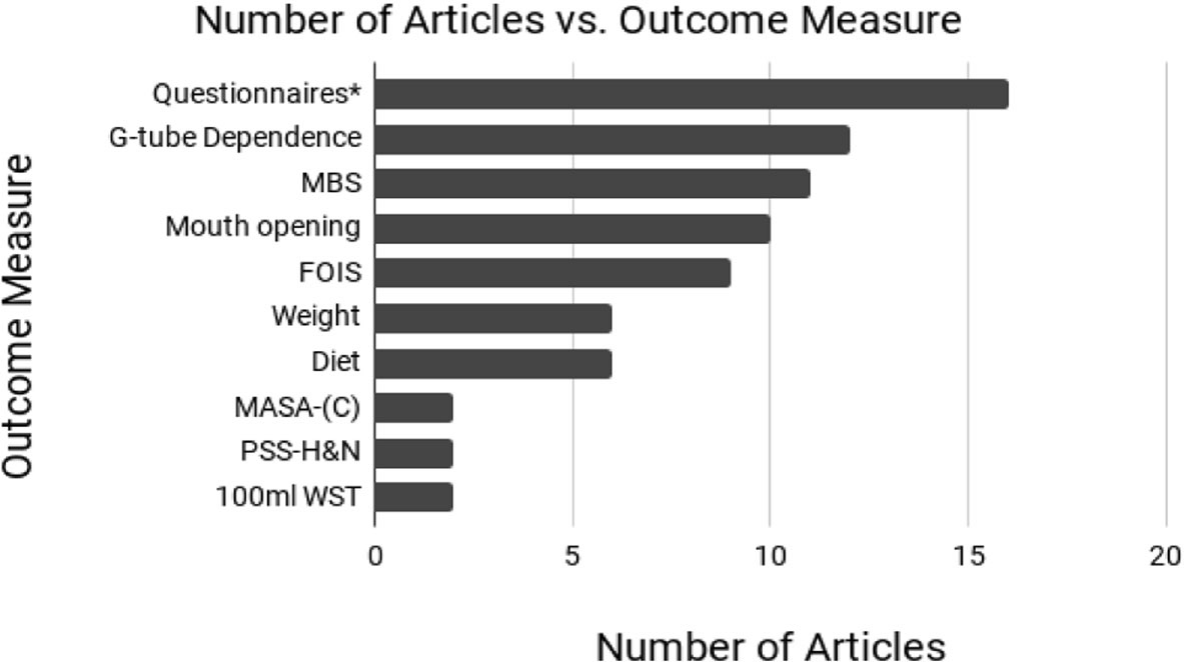
Number of articles that included specific outcome measures in their prehabilitation program. *Questionnaires included validated questionnaires such as the EORTC-30, EORTC H&N35, HADS, MDADI, DHI, VHI, as well as study specific questionnaires. *Abbreviations:* MBS = Modified Barium Swallow; FOIS = Functional Oral Intake Scale; MASA-(C) = Mann Assessment of Swallowing Ability – Cancer; PSS-H&N = Performance Status Scale for Head and Neck Cancer Patients; WST = Water Swallow Test. *Outcome measures that were reported by a single article were not included in the Figure. These are:* Accusens T Taste function kit, Functional Intra-oral Glasgow Scale, length of hospital stay, LENT/SOMA scale (categorizes severity of trismus based on interincisor distance and impact on swallowing), max inspiratory and expiratory pressures, max isometric tongue pressure, maximal tongue strength (IOPI), Myrin goniometer (range of mobility of H&N), oromotor, toxicity (OCA, CTCAE), Patient Generated Subjective Global Assessment (PGSGA45), peak cough flow, salivation, University of Pennsylvania Smell Identification Test, and the Volume-Viscosity Swallow Test

The types of exercises included in the reviewed studies were as varied as the outcome measures used to assess the effectiveness of these exercises. Figure 4 illustrates the top 10 outcome measures used. A number of validated tools and study-specific questionnaires were included across the studies. Ahlberg [14] measured Quality of Life outcomes using the European Organization for Research and Treatment of Cancer Quality of Life Questionnaire C30 (EORTC QLQ-C30), European Organization for Research and Treatment of Cancer Quality of Life Questionnaire Head and Neck Module (EORTC QLQ-H&N35), Hospital Anxiety and Depression Scale (HADS), and a program specific questionnaire. Kulbersh [17] used the M. D. Anderson Dysphagia Inventory (MDADI) and van der Molen [20] used a structured, study-specific questionnaire for quality of life evaluation. Carmignani [30] used the EORTC QLQ-C30, EORTC QLQ-H&N35, Dysphagia Handicap Index (DHI), MDADI, and Voice Handicap Index (VHI). While Carnaby-Mann et al. [15] indicate that quality of life questionnaires were used, the specific questionnaires used were not explicitly stated in the methods.

Only one article [37] compared prehabilitation using the Shaker versus the Mendelsohn maneuver and found superior results in the Shaker group, including improvements in hyoid bone movement, thyrohyoid shortening, upper esophageal sphincter opening, reduced aspiration score and reduced feeding tube rates. Four articles included in the present review were proposed protocols or feasibility studies reporting no outcomes [22, 26, 29, 32]. Of the remaining studies that reported on outcomes, 20 studies reported on short term outcomes (12 months or less), while five reported on outcomes beyond 12 months. One article specifically reported outcomes as a 6 year follow-up [23]. The majority of the articles reported positive outcomes as a result of prehabilitation. However, two articles included in this review found no difference or negative outcomes from prehabilitation. Mortensen et al. [18] found no significant difference in their dysphagia outcomes between the prehabilitation group and the control group. They did report a trend for consistently better scores in the prehabilitation group, though the experimental group was small. Ahlberg et al. [14] found no significant difference between the prehabilitation group and the control group on their primary measures of weight loss and two-year survival. The authors also found no significant difference on their secondary measures of quality of life and anxiety/ depression. In addition, Ahlberg et al. [14] found that the patients in the prehabilitation group had significantly worse results on some of their secondary outcome measures, including sick leave and self-reported loss of function. It should be noted that the dose for Mortensen et al.’s [18] prehabilitation program was 210, while that of Ahlberg and colleagues [14] was 440.

When we take a closer look at control groups used or proposed in the articles reviewed, variability exists once again. The most common control group was a “usual care” group (10 articles) [15, 16, 18–20, 25, 29, 30, 33, 41]. Two articles created control groups based on compliance, such that those participants that were noncompliant became the control group [36, 42]. Two articles define the control group as the group of patients that were seen prior to the implementation of a prehabilitation program [38, 40]. Two articles had control groups based on the mode of service delivery [22, 32]. Three articles had control groups that received exercise based therapy post cancer treatment [17, 26, 35]. Additional control groups included sham (buccal) exercises [15], swallowing practice as opposed to an exercise program [21], an alternate RT facility [14], and a group that received treatment immediately when dysphagia developed [37]. Seven articles did not include control groups, [22–24, 27, 31, 34, 39] and one article indicates a control group used but does not define the control group [28].

## Discussion

With an increased interest in programs used to prevent or limit impairment that results from HNC treatments comes a need to understand the specifics of such programs. The aim of this review was to assist with the design of a prehabilitation program for HNC patients. This review revealed a lack of consensus on the definition of prehabilitation (i.e., when exercises are introduced in the continuum of care), the type and dose of recommended exercises, and treatment duration. Furthermore, as the articles included in this review used a variety of outcomes measures, it was difficult to discern if some prehabilitation programs provided more favorable outcomes than others.

First and foremost, it would be beneficial for researchers in this field to qualify the definition of *prehabilitation,* as stated previously, by using either *pretreatment prehabilitation* or *treatment concurrent prehabilitation,* or to reach a consensus on the general use of the term. With respect to recommended exercise types, some programs focused on a multipronged approach, such as exercises for trismus, tongue range of motion, neck range of motion and swallow-specific exercises, while others limited their exercises to only swallow-specific exercises. The most common swallow-specific exercises used were the Mendelsohn maneuver, followed by the effortful swallow, Shaker, and Masako maneuver. Whereas these exercises all have some level of evidence to support their use in swallowing rehabilitation, it was unclear from the current literature as to which exercise was most effective, if they were all equally effective, or if there was an ideal combination of exercises that provide the best outcomes.

With respect to the dose (number of exercises, number of repetitions for each exercise, frequency of exercises), the most common recommendations appeared to be ten repetitions of an exercise, three times per day [16, 18, 27, 36, 37, 40]. As most articles did not provide a justification for this dose, it was unclear if this regimen was in fact the ideal dose or if it was used as a round number for patients to remember and support adherence. The lack of a standardized dosage within dysphagia exercise programs has become more apparent as these programs increase in popularity. A recent scoping review by Krekeler and colleagues [43] sheds additional light on this topic. Only three articles [20, 21, 37] provided justification for the dose chosen in their prehabilitation programs. According to Burkhead et al., [9] the ideal dose must be selected depending on the end goal of the exercise program: building strength, endurance or both. However, clinicians are cautioned against blindly applying values derived from exercise science to dysphagia therapy as muscles involved in mastication and swallowing exhibit unique fiber types, architecture, and composition, unlike any other human skeletal muscle [9]. It is currently unknown if the muscles involved in mastication and swallowing respond in a similar fashion to strength training as skeletal muscle groups.

When discussing recommended exercise dose, adherence to that regimen is an additional important consideration in the design of a prehabilitation program. Articles such as Hutcheson et al. [42] and Shinn et al. [39] have found superior results when adherence was high. A number of studies in this review reported on adherence to home swallowing programs [18, 22, 24, 27, 31, 33, 34, 39, 40, 42]. One barrier to adherence may be the total number of exercises required in the day (i.e. daily dose) [44]. Clinicians should recommend a prehabilitation program that balances the number of exercises required to create functional gains with one that patients can complete successfully.

The duration of treatment ranged from the duration of RT up to 1 year after, with the most common duration defined as the duration of RT treatment. With respect to principles of neural plasticity, the duration of treatment is important and should be tailored with the effects of program intensity (to push the system beyond typical use) and detraining in mind as significant decreases in strength gains can be observed after approximately 4 weeks following cessation of training [9].

Outcome tools noted in this review ranged from subjective measures such as patient reported outcomes and clinician-based measures, to objective measures such as weight and mouth opening. Specific clinical end-points such as overall survival and presence of a g-tube were also used. Tools such as questionnaires, mouth opening and presence of a g-tube were more predominant in this review likely because they tend to be easier to administer and are readily available. Objective assessments such as the Modified Barium Swallow (MBS), Fiberoptic Endoscopic Evaluation of Swallowing (FEES), or Magnetic Resonance Imaging (MRI) were used less frequently, likely due to reduced accessibility, increase in potential patient risk, and expertise and cost required for administering and interpreting these measures. Functional outcomes in future studies should consider a multimodal approach to assessment (i.e., use outcomes that address patient perspectives, clinician opinion, and objectives measures). The outcome measures selected should also be sensitive to the goals of the program. For example, if the prehabilitation program exercises are limited to preventing trismus, outcomes should be related to mouth opening rather than swallowing. Finally, outcome measures should address if and how functional changes impact patient quality of life.

The majority of articles (18 of 19) reviewed reported positive outcomes as a result of prehabilitation programs. While these articles were challenging to compare due to the variety of prehabilitation programs offered and disparity in outcome tools used, general consensus does support the use of prehabilitation programs. It is possible that the variation found in functional outcomes is in fact a function of the variation in outcome tools used. The variation may also be a result of the variation in control groups. When we look at control groups, the use of a “usual care” group was the most common control group. An article by Carnaby and Harenberg [45] found that the concept of “usual care” for dysphagia is not supported due to the lack of uniformity in the treatment schemes and strategies used by SLPs to treat dysphagic patients. While usual care was described in some articles, it was not described in all of the articles. When usual care was described, variations also existed. While usual care at some centers comprises of referral and treatment of dysphagia as it develops [15, 16, 18, 29, 30], others include some exercises but these are not monitored [25, 33]. It is recognized that it is challenging to create a suitable control group. Retrospective analysis with historical controls can introduce issues including selection bias, recall bias, and confounds such as cancer treatment advances. Compliance bias is a well-known issue with simple comparisons between compliant versus noncompliant patients. Prospective trials would require controls that are appropriately managed for demographic, tumor, and treatment characteristics. Another challenge with the control groups is demonstrated by the lack of consensus with the definition “prehabilitation.” Kulbersh and colleagues [17] used a study design in which the experimental group began their prehabilitation exercises 2 weeks prior to the initiation of CRT, and their control group, defined as “post-treatment” began their exercise program at the onset of CRT. This presents a challenge when comparing studies as within this review, it was found that the onset of CRT is defined as the “prehabilitation” group in two thirds of the articles reviewed.

Articles reviewed herein included participants with a mix of primary cancer sites such as nasopharynx, oral cavity, pharyngeal, laryngeal, or unknown primary sites. Hence, it was unclear if one exercise has a positive impact for all HNC patients regardless of primary site, or if specific exercises have the most impact based on tumor site. Furthermore, treatment modality preference at different cancer centers provided another source of variation. All articles included patient participants undergoing RT or concurrent CRT. Six articles [22, 27, 29, 30, 33, 34] included surgical patients; however, the prehabilitation program appeared to be implemented preRT/CRT, not pre-surgery. No articles looked at the impact of a prehabilitation program on surgical HNC patients, though this is common practice in a number of surgical procedures such as orthopedic surgery and is recommended by the American College of Surgeons [46].

This article does not address other aspects of prehabilitation described by Silver and colleagues who recommended that cancer prehabilitation include “physical and psychological assessments that establish a baseline functional level, identify impairments, and provide interventions that promote physical and psychological health to reduce the incidence and/or severity of future impairments” [5]. Silver et al. [6] discussed how clinicians must target oncological services as a global system and go beyond function-specific exercises. In other words, a good prehabilitation program should target nutrition, mental health, and physical health, rather than focus exclusively on swallowing function.

Since the start of this literature review, new clinical trials targeting prehabilitation in this patient population were registered, which holds promise in elucidating some of the challenges presented by the results of this review. These include a trial registered under Wessel and colleagues where 240 patients were randomized to the prehabilitation or standard of care. Prehabilitation consisted of mouth opening and swallowing exercises (half an hour, three times a week, 5 to 6 weeks) and progressive resistance training with a physiotherapist (twice a week), as well as a home-program that begins at the start of RT and continues beyond the length of cancer treatment. Another registered trial is from Toronto’s University Health Network, where an anticipated 952 patient participants are randomized to three arms: RE-ACTIVE (described as intervention provided once dysphagia is diagnosed), PRO-ACTIVE EAT (described as early, low intensity intervention before the start of RT), and PRO-ACTIVE EAT and EXERCISE (described as early, high intensity proactive intervention before the start of RT).

This was a state-of-the-art review and it is possible that a systematic review may have identified additional studies. For example, this review used free text searches rather than MeSH terms. However, the ultimate goal of this work was to offer knowledge users (clinicians and researchers) timely yet still accurate and current information on the topic; a State-of-the-Art review does this by summarizing what is known, offering new points of view on the topic, and suggesting priorities for future investigation on current topics [13]. A State-of-the-Art review can influence both, practice and future research in a timely fashion, particularly given the concerns this work has highlighted with respect to what constitutes prehabilitation and when in the continuum of care it is offered. Another limitation was the authors’ attempt to represent unique protocols for prehabilitation. Therefore, where articles appeared to report on the same patient cohort, only one article was included. For example, if multiple articles were published on the same cohort, one on prehabilitation outcomes and one on adherence to prehabilitation, only the article reporting on outcomes was included. With these criteria, it is possible that some articles were missed or some protocols were included twice.

## Conclusion

Consensus is lacking across most areas related to prehabilitation in HNC. It is difficult to develop an optimal prehabilitation program as this review of the literature, similar to that of general dysphagia literature, [47] has shed light on the variability of prehabilitation timing, exercise type, dose, duration of treatment, and outcomes associated with prehabilitation. The authors of the present review recommend starting with exercises that address anticipated function loss at the most common recommended regimen of ten repetitions, three times a day, and supplementing these exercises with increased physical activity as well as consultation on nutrition, and mental health. The program should begin as close to cancer diagnosis as possible and be followed by early rehabilitation. Future research should focus on reaching a consensus on the definition of prehabilitation, on outcome measures sensitive to the goals of the program, and on designing programs that will optimize adherence and functional outcomes.

## Data Availability

Not applicable.

## Abbreviations

CRT: Chemoradiation
CTCAE: Common Terminology Criteria for Adverse Events
DHI: Dysphagia Handicap Index
EORTC QLQ-C30: European Organization for Research and Treatment of Cancer Quality of Life Questionnaire C30
EORTC QLQ-H&N35: European Organization for Research and Treatment of Cancer Quality of Life Questionnaire Head and Neck Module
FEES: Fiberoptic Endoscopic Evaluation of Swallowing
FOIS: Functional Oral Intake Scale
HADS: Hospital Anxiety and Depression Scale
HNC: Head and Neck Cancer
HPV: Human Papillomavirus
IOPI: Iowa Oral Performance Instrument
MASA-(C): Mann Assessment of Swallowing Ability Cancer
MBS: Modified Barium Swallow
MDADI: M. D. Anderson Dysphagia Inventory
MRI: Magnetic Resonance Imaging
OCA: Oral Cavity Assessment
PGSGA45: Patient Generated Subjective Global Assessment
PSS-H&N: Performance Status Scale for Head and Neck Cancer Patients
RT: Radiation Therapy
SLP: Speech-Language Pathologist
SSG: Super Supraglottic Swallow
VHI: Voice Handicap Index
WST: Water Swallow Test
